# The relationship between triglyceride-glucose index and albuminuria in United States adults

**DOI:** 10.3389/fendo.2023.1215055

**Published:** 2023-08-23

**Authors:** Zhaoxiang Wang, Han Qian, Shao Zhong, Tian Gu, Mengjiao Xu, Qichao Yang

**Affiliations:** ^1^ Department of Endocrinology, Affiliated Kunshan Hospital of Jiangsu University, Kunshan, Jiangsu, China; ^2^ Department of Cardiology, Affiliated Taicang Hospital of Soochow University, Suzhou, Jiangsu, China; ^3^ Department of Endocrinology, Affiliated Wujin Hospital of Jiangsu University, Changzhou, Jiangsu, China

**Keywords:** triglyceride-glucose index, insulin resistance, metabolic syndrome, albuminuria, NHANES

## Abstract

**Purpose:**

Triglyceride-glucose (TyG) index is a simple and reliable indicator of metabolic dysfunction. We aimed to investigate a possible relationship between TyG index and albuminuria in the United States adult population.

**Methods:**

This cross-sectional study was conducted among adults with complete TyG index and urinary albumin/urinary creatinine (UACR) from 2011-2018 National Health and Nutrition Examination Survey (NHANES). The independent relationship between TyG index and albuminuria (UACR>30mg/g) was evaluated. TyG index was compared with insulin resistance represented by homeostatic model assessment of insulin resistance (HOMA-IR), and metabolic syndrome. Subgroup analysis was also performed.

**Results:**

A total of 9872 participants were included in this study, and the average TyG index was 8.53 ± 0.01. The proportion of albuminuria gradually increased with the increase of TyG index quartile interval. Elevated TyG index was independently associated with albuminuria, and this association persisted after additional adjustments for HOMA-IR or dichotomous metabolic syndrome. The area under the ROC curve (AUC) of TyG index was larger than that of log (HOMA-IR). Subgroup analysis suggested that the relationship between TyG index and albuminuria is of greater concern in age<60, overweight/obese, diabetic, and metabolic syndrome patients.

**Conclusion:**

The TyG index may be a potential epidemiological tool to quantify the role of metabolic dysfunction, rather than just insulin resistance, in albuminuria in the United States adult population. Further large-scale prospective studies are needed to confirm our findings.

## 1 Introduction

As a progressive disease, chronic kidney disease (CKD) has become one of the leading causes of death and suffering in the 21st century, affecting more than 10% of the general population worldwide (1). Patients with CKD also present with an increased risk of cardiovascular disease (2). In normal conditions, albumin is excreted in minimal amounts in the urine, making its detection challenging. However, when the glomerular filtration membrane is disrupted, the proteins in the glomerular filtration fluid are increased. In the early stages of glomerulopathy, when a routine urine protein test is negative, urinary microalbumin levels may vary and increase as the disease progresses (3). It is the most sensitive and reliable diagnostic index for early diabetic nephropathy and early hypertensive nephropathy (4, 5). Elevated albuminuria levels are an independent predictor of cardiovascular risk and have shown a strong association with non-alcoholic fatty liver disease (NAFLD) (6–10). Albuminuria is present in up to 40% of diabetic patients and has become an essential screening program for diabetic kidney disease (DKD) and diabetic cardiovascular events (11, 12).

Insulin resistance and metabolic syndrome are strongly associated with albuminuria (13–15). The HOMA-IR index was obtained using the homeostatic model assessment of insulin resistance (16). It is widely utilized in research on the development and progression of metabolic diseases, acting as a commonly employed indicator to evaluate the severity of insulin resistance (17, 18). Metabolic syndrome, defined by a combination of several laboratory and physical examination measures, is a dichotomous approach and thus does not allow for an assessment of the degree of risk (19). TyG index is a measure of metabolic dysfunction calculated from fasting triglyceride (TG, mg/dL) and fasting plasma glucose (FPG, mg/dL) measurements. It was considered a reliable surrogate marker of insulin resistance (20, 21). Multiple studies have indicated that TyG and HOMA-IR exhibit comparable accuracy in measuring insulin resistance when compared to the gold standard hyperinsulinemic-euglycemic clamp (19, 22, 23). Moreover, TyG index performed better than HOMA-IR in the diagnosis of metabolic syndrome (19, 24). NAFLD is a further expression of the metabolic syndrome (25). TyG index also serves as an effective surrogate marker for NAFLD and is associated with the underlying mechanisms of the disease, as well as NAFLD-related pathology, including extrahepatic tumors (26). Previous studies have demonstrated elevated TyG index can predict the progression of coronary artery calcification and the occurrence of adverse cardiovascular events (20, 27).

While several studies have investigated the association between TyG index and albuminuria, most of these studies have been limited to a single population and few have involved the United States population. More importantly, none of these studies considered the effects of insulin resistance and metabolic syndrome (28–31). The purpose of this study was to investigate the association between TyG index and albuminuria in the population of the National Health and Nutrition Examination Survey (NHANES) based on larger sample size. TyG index was compared vs. insulin resistance represented by HOMA-IR, and vs. metabolic syndrome.

## 2 Materials and methods

### 2.1 Data source

All the data in this study came from NHANES, which is a research program designed to assess the health and nutrition status of adults and children in the United States administered by the National Center for Health Statistics (NCHS) (32). NHANES has a complex, multistage, probability sampling design that allows it to be generalized to the US population (32). The protocol for the NHANES study was approved by the NCHS Research Ethics Review Board, and its detailed design and data are publicly available at https://www.cdc.gov/nchs/nhanes/. The current study included all participants older than 20 years with complete TyG index, albuminuria, and glomerular filtration rates from NHANES 2011 to 2018 who were in the fasting subsample.

### 2.2 Outcome definitions

Urinary albumin and creatinine were measured by solid-phase fluorescent immunoassay and modified Jaffe kinetic method, respectively. UACR was calculated by dividing the urinary albumin (mg/dL) concentration by the urinary creatinine concentration (mg/L). UACR was categorized as <30, 30–300, and ≥300mg/g in subgroup analysis. Albuminuria, defined as an UACR>30 mg/g, was considered as an outcome variable in our analysis (33). UACR 30–300 and ≥ 300mg/g correspond to moderately increased albuminuria and severely increased albuminuria, respectively.

### 2.3 Exposure definitions

TyG index was calculated as ln (fasting triglyceride[mg/dl] × fasting plasma glucose[mg/dl]/2). HOMA-IR was calculated using the following formula: (fasting insulin[µIU/ml] × fasting plasma glucose[mmol/L])/22.5. HOMA-IR was log-transformed for modeling in this study. Metabolic syndrome is defined based on criteria developed jointly by international organizations such as the International Diabetes Federation, the National Heart, Lung and Blood Institute and the American Heart Association (34). Metabolic syndrome includes at least three of the five: elevated waist circumference (WC, cm), hypertension, hyperglycemia, elevated TG and decreased high density lipoprotein cholesterol (HDL-c, mg/dl).

### 2.4 Covariate definitions

Demographic data (age, gender, and race) was obtained. Some potential covariates were also included in this study, such as smoking status (never/former/current), physical activity (vigorous/moderate/less than moderate), hypertension (yes/no), diabetes (yes/no), cardiovascular disease (yes/no), body mass index (BMI, kg/m^2^), WC, total cholesterol (TC, mg/dl), HDL-c, low density lipoprotein cholesterol (LDL-c, mg/dl), serum creatinine (Scr, mg/dl), and estimated glomerular filtration rate (eGFR, ml/min/1.73 m^2^). BMI was categorized as <25, 25-29.9 and ≥30 kg/m^2^, which corresponded to normal weight, overweight, and obese population for participants. The presence of cardiovascular disease was determined based on self-reported history of heart attack, stroke, congestive heart failure, coronary artery disease, or angina. eGFR was calculated according to the CKD Epidemiology Collaboration (CKD-EPI) creatinine equation consisting of age, gender, race, and Scr (35). Detailed measurement procedures for all variables in this study were publicly available in the NHANES database.

### 2.5 Statistical analysis

All statistical analyses were performed in accordance with Centers for Disease Control and Prevention (CDC) guidelines. A complex multistage cluster survey design was considered, and fasting subsample weights combined with four cycles were applied. Continuous variables were expressed as weighted mean with standard error (SE), and categorical parameters were presented as proportions. Weighted one-way ANOVA and weighted chi-squared tests were used for multiple-group comparisons of continuous and categorical variables respectively. Logistic and linear models were used to test the association between TyG index (continuous/quartile) and albuminuria and UACR in different models. The variance inflation factor (VIF) was employed to assess the level of collinearity among independent variables (36). The VIF is computed using a multivariate linear regression model. The VIF of each independent variable is equal to 1 divided by (1 − R^2^), where R^2^ represents the coefficient of determination obtained by regressing the independent variables on the remaining independent variables. Independent variables with VIF greater than 5 were excluded. In model 1, no covariates were adjusted. In model 2, age, gender, and race were adjusted. Model 3 was adjusted for age, gender, race, smoking status, physical activity, hypertension, diabetes, cardiovascular disease, BMI, Scr, and eGFR. Weighted Pearson correlation analysis was used to evaluate the correlation between TyG index and other parameters. ROC was used to compare TyG index and TyG-derived indices with log (HOMA-IR). Age (<60/≥60 years), gender (female/male), BMI (normal weight/overweight/obese), diabetes (yes/no), hypertension (yes/no), metabolic syndrome (yes/no), cardiovascular disease (yes/no), and eGFR (<60/60-90/≥90ml/min/1.73 m^2^) were stratified for subgroup analysis. Empower software (http://www.empowerstats.com) and R version 4.1.0 (http://www.R-project.org) were employed for all analyses. ***P*** value <0.05 was considered statistically significant.

## 3 Results

### 3.1 Baseline characteristics of study population

According to the inclusion criteria, a total of 9872 subjects were included in the study, with an average age of 46.81 ± 0.35 years, including 48.94% male. We compared general information and clinical indicators for subjects in the non-albuminuria, moderately increased albuminuria and severely increased albuminuria groups (**Table 1**). Compared with non-albuminuria group, age, male, smoking proportion, less than moderate physical activity, hypertension prevalence, diabetes prevalence, cardiovascular disease prevalence, metabolic syndrome prevalence, BMI, WC, FPG, FIns, TG, Scr, urinary albumin, and HOMA-IR were significantly increased in moderately increased albuminuria and severely increased albuminuria groups (***P*** <0.001), and LDL-c, HDL-c, urinary creatinine, and eGFR were decreased (***P*** <0.05). There were also differences in race distribution among groups (***P*** <0.001). Note that TyG levels were higher in the moderately increased albuminuria and severely increased albuminuria groups than in the non-albuminuria group (***P*** <0.001).

**Table 1:** Baseline characteristics of study population according to UACR, weighted.

	Overall	Non-albuminuria	Moderately increased albuminuria	Severely increased albuminuria	P value
Age (year)	46.81 ± 0.35	45.93 ± 0.36	54.21 ± 0.96	58.30 ± 1.34	<0.001
Male gender, % (SE)	48.94 (0.57)	49.49 (0.63)	42.12 (2.06)	53.11 (4.25)	<0.001
Race, % (SE)					<0.001
Mexican American	8.99 (0.95)	8.72 (0.94)	11.17 (1.45)	13.05 (2.99)	
Other Hispanic	11.31 (0.98)	10.93 (0.95)	13.93 (1.64)	19.36 (2.96)	
Non-Hispanic White	64.40 (1.75)	65.01 (1.72)	61.07 (2.76)	47.37 (4.81)	
Non-Hispanic Black	6.39 (0.65)	6.42 (0.66)	5.59 (0.93)	9.22 (2.51)	
Other Races	8.90 (0.58)	8.92 (0.59)	8.23 (0.89)	11.00 (2.15)	
Smoking status, % (SE)					<0.001
Never	57.31 (0.97)	58.04 (0.97)	51.33 (2.32)	46.67 (3.98)	
Former	24.49 (0.78)	23.92 (0.78)	29.82 (2.29)	29.33 (3.40)	
Current	18.20 (0.82)	18.04 (0.86)	18.85 (2.19)	24.00 (4.43)	
Physical activity, % (SE)					<0.001
Vigorous	22.85 (0.72)	23.30 (0.76)	18.85 (1.97)	17.71 (3.74)	
Moderate	23.79 (0.66)	24.19 (0.75)	21.19 (1.56)	14.52 (2.80)	
Less than moderate	53.36 (0.88)	52.51 (0.97)	59.95 (2.29)	67.77 (3.96)	
Hypertension, % (SE)	32.55 (0.87)	29.98 (0.85)	52.84 (2.35)	73.71 (4.09)	<0.001
Diabetes, % (SE)	9.88 (0.46)	7.73 (0.41)	25.23 (1.98)	52.78 (4.43)	<0.001
Cardiovascular disease, % (SE)	8.90 (0.43)	7.45 (0.42)	19.16 (1.47)	37.98 (3.79)	<0.001
Metabolic syndrome, % (SE)	34.60 (0.81)	32.34 (0.85)	53.29 (2.19)	66.23 (3.70)	<0.001
BMI (kg/m^2^)	29.16 ± 0.14	29.02 ± 0.15	30.38 ± 0.37	31.16 ± 0.93	<0.001
WC (cm^2^)	99.47 ± 0.36	99.01 ± 0.36	103.08 ± 0.84	106.65 ± 2.31	<0.001
FPG (mg/dL)	107.22 ± 0.47	104.88 ± 0.40	123.93 ± 1.96	153.55 ± 6.08	<0.001
FIns (µIU/ml)	12.87 ± 0.23	12.26 ± 0.20	17.40 ± 1.10	24.56 ± 4.45	<0.001
TG (mg/dL)	117.44 ± 1.71	115.03 ± 1.79	133.36 ± 4.50	172.31 ± 16.20	<0.001
TC (mg/dL)	189.27 ± 0.76	189.25 ± 0.81	189.69 ± 1.75	188.45 ± 3.65	0.930
LDL-c (mg/dL)	111.93 ± 0.58	112.24 ± 0.61	109.19 ± 1.61	108.34 ± 3.43	0.030
HDL-c (mg/dL)	54.24 ± 0.32	54.29 ± 0.31	54.48 ± 1.03	49.81 ± 1.51	0.004
Scr (mg/dl)	0.87 ± 0.00	0.86 ± 0.00	0.90 ± 0.03	1.52 ± 0.12	<0.001
eGFR (ml/min/1.73 m^2^)	90.67 ± 0.51	91.11 ± 0.53	89.80 ± 1.51	70.16 ± 3.65	<0.001
Urinary albumin (mg/L)	37.13 ± 2.66	9.96 ± 0.14	86.51 ± 3.65	1334.68 ± 114.73	<0.001
Urinary creatinine (mg/dl)	127.94 ± 1.41	129.31 ± 1.55	116.96 ± 2.98	107.25 ± 5.02	<0.001
HOMA-IR	3.72 ± 0.08	3.39 ± 0.06	5.93 ± 0.45	10.74 ± 2.15	<0.001
TyG index	8.53 ± 0.01	8.50 ± 0.01	8.76 ± 0.04	9.10 ± 0.08	<0.001

BMI, body mass index; WC, Waist circumference; FPG, fasting plasma glucose; FIns, fasting plasma insulin; TG, triglyceride; TC, total cholesterol; LDL-c, low-density lipoprotein cholesterol; HDL-c, high-density lipoprotein cholesterol; SCr, serum creatinine; eGFR, estimated glomerular filtration rate; HOMA-IR, homeostatic model assessment of insulin resistance; TyG index, Triglyceride-glucose (TyG) index.

### 3.2 Clinical features of the participants according to the quartiles of TyG index

According to the TyG level of all subjects, they were divided into four groups from low to high: quartile I (TyG<8.07), quartile II (8.07<TyG ≤ 8.50), quartile III (8.50<TyG ≤ 8.96), and quartile IV (TyG>8.96) (**Table 2**). Compared with quartile I-TyG group, age, male, smoking proportion, hypertension prevalence, diabetes prevalence, cardiovascular disease prevalence, metabolic syndrome prevalence, BMI, WC, FPG, FIns, TG, TC, Scr, urinary albumin, and HOMA-IR were significantly increased in quartile II-TyG, quartile III-TyG, and quartile IV-TyG groups increased significantly (***P*** <0.001), and LDL-c, HDL-c, and eGFR decreased significantly (***P*** <0.001). Most importantly, with the gradual increase of TyG level, the UACR level (19.34 ± 2.44 vs. 21.88 ± 2.94 vs. 25.75 ± 2.59 vs. 72.46 ± 10.90, ***P*** <0.001) and the number of people with albuminuria (7.13% vs. 7.66% vs. 8.27% vs. 16.58%, ***P <*** 0.001) gradually increased.

**Table 2:** Clinical and laboratory characteristics based on TyG index quartiles, weighted.

	Quartile I	Quartile II	Quartile III	Quartile IV	P value
Age (year)	40.19 ± 0.57	46.12 ± 0.50	49.33 ± 0.49	51.93 ± 0.45	<0.001
Male gender, % (SE)	39.59 (1.32)	46.99 (1.26)	51.80 (1.28)	57.95 (1.42)	<0.001
Race, % (SE)					<0.001
Mexican American	6.90 (0.95)	8.13 (0.98)	10.05 (1.06)	11.02 (1.16)	
Other Hispanic	18.50 (1.63)	12.32 (1.09)	8.05 (0.78)	6.00 (0.68)	
Non-Hispanic White	59.65 (2.19)	64.53 (1.97)	65.90 (1.96)	67.77 (1.76)	
Non-Hispanic Black	6.06 (0.86)	6.30 (0.69)	6.90 (0.70)	6.32 (0.85)	
Other Races	8.89 (0.87)	8.73 (0.71)	9.10 (0.76)	8.88 (0.76)	
Smoking status, % (SE)					<0.001
Never	65.40 (1.56)	60.26 (1.85)	54.11 (1.39)	48.95 (1.44)	
Former	20.39 (1.32)	21.91 (1.35)	26.58 (1.30)	29.35 (1.28)	
Current	14.20 (1.00)	17.84 (1.40)	19.31 (1.23)	21.70 (1.15)	
Physical activity, % (SE)					0.683
Vigorous	23.68 (1.45)	22.65 (1.40)	22.01 (1.14)	23.05 (1.20)	
Moderate	24.13 (1.24)	23.60 (1.24)	23.28 (1.15)	24.17 (1.29)	
Less than moderate	52.19 (1.51)	53.75 (1.69)	54.72 (1.36)	52.78 (1.46)	
Hypertension, % (SE)	18.90 (1.08)	27.48 (1.37)	37.24 (1.36)	47.49 (1.81)	<0.001
Diabetes, % (SE)	1.96 (0.35)	4.52 (0.50)	8.13 (0.69)	25.83 (1.43)	<0.001
Cardiovascular disease, % (SE)	4.69 (0.52)	7.71 (0.68)	10.09 (0.77)	13.40 (0.94)	<0.001
Metabolic syndrome, % (SE)	7.10 (0.76)	17.57 (1.00)	33.60 (1.24)	82.87 (1.31)	<0.001
BMI (kg/m^2^)	26.33 ± 0.21	28.25 ± 0.20	30.17 ± 0.22	32.08 ± 0.21	<0.001
WC (cm^2^)	90.76 ± 0.52	96.95 ± 0.51	102.54 ± 0.48	108.18 ± 0.52	<0.001
FPG (mg/dL)	94.82 ± 0.32	100.23 ± 0.33	105.18 ± 0.46	129.93 ± 1.34	<0.001
FIns (µIU/ml)	7.66 ± 0.22	10.49 ± 0.28	13.74 ± 0.35	20.03 ± 0.57	<0.001
TG (mg/dL)	49.94 ± 0.39	81.01 ± 0.38	118.78 ± 0.70	226.33 ± 4.23	<0.001
TC (mg/dL)	171.65 ± 1.00	185.28 ± 1.05	195.27 ± 0.97	205.95 ± 1.20	<0.001
LDL-c (mg/dL)	97.90 ± 0.90	111.53 ± 0.87	119.85 ± 0.81	118.99 ± 0.92	<0.001
HDL-c (mg/dL)	63.79 ± 0.55	57.54 ± 0.50	51.66 ± 0.32	43.28 ± 0.31	<0.001
Scr (mg/dl)	0.83 ± 0.01	0.88 ± 0.01	0.88 ± 0.01	0.90 ± 0.01	<0.001
eGFR (ml/min/1.73 m^2^)	96.04 ± 0.78	89.96 ± 0.71	88.79 ± 0.66	87.65 ± 0.73	<0.001
Urinary albumin (mg/L)	22.97 ± 3.69	23.89 ± 2.70	28.57 ± 3.29	75.17 ± 11.03	<0.001
Urinary creatinine (mg/dl)	128.70 ± 2.65	125.85 ± 2.56	128.33 ± 2.32	128.89 ± 2.18	0.5275
UACR (mg/g)	19.34 ± 2.44	21.88 ± 2.94	25.75 ± 2.59	72.46 ± 10.90	<0.001
Albuminuria, % (SE)	7.13 (0.64)	7.66 (0.64)	8.27 (0.71)	16.58 (1.08)	<0.001
HOMA-IR	1.83 ± 0.06	2.69 ± 0.11	3.67 ± 0.11	6.86 ± 0.25	<0.001

### 3.3 Association of TyG index with albuminuria independent of HOMA-IR or metabolic syndrome

Our results showed that higher TyG index was associated with increased likelihood of increased albuminuria. This association was significant in model 1 [OR (95%CI): 1.871 (1.725-2.030), ***P*** <0.001], model 2 [OR (95%CI): 1.772 (1.622-1.936), ***P*** <0.001], model 3 [OR (95%CI): 1.391 (1.258-1.539), ***P*** <0.001], and the model with additional adjustment for log (HOMA-IR) [OR (95%CI): 1.346 (1.204-1.506), ***P*** <0.001] or metabolic syndrome [OR (95%CI): 1.316 (1.178-1.470), ***P*** <0.001]. We transformed TyG index from a continuous variable to a categorical variable. Compared to the lowest TyG index quartile, the highest TyG index quartile had 32.0% and 27.3% increased risk of albuminuria in the model with additional adjusted for log (HOMA-IR) or metabolic syndrome, respectively (**Table 3**). Using the UACR as a dependent variable for the linear regression analysis, we can also find that TyG index is closely related to UACR (**Table 4**).

**Table 3:** The association between TyG index and albuminuria.

Albuminuria	OR (95%CI) P value				
Continuous	Model 1	Model 2	Model 3	additionally adjusted for log (HOMA-IR)	additionally adjusted for metabolic syndrome
TyG index	1.871 (1.725, 2.030) <0.001	1.772 (1.622, 1.936) <0.001	1.391 (1.258, 1.539) <0.001	1.346 (1.204, 1.506) <0.001	1.316 (1.178, 1.470) <0.001
Categories					
Quartile I	1.00	1.00	1.00	1.00	1.00
Quartile II	1.317 (1.087, 1.597) 0.005	1.101 (0.902, 1.344) 0.342	1.024 (0.832, 1.260) 0.823	0.993 (0.806, 1.224) 0.950	1.005 (0.816, 1.237) 0.966
Quartile III	1.451 (1.201, 1.753) <0.001	1.139 (0.934, 1.390) 0.197	0.953 (0.772, 1.175) 0.650	0.902 (0.728, 1.118) 0.348	0.902 (0.728, 1.117) 0.344
Quartile IV	2.856 (2.401, 3.397) <0.001	2.246 (1.866, 2.705) <0.001	1.463 (1.189, 1.800) <0.001	1.320 (1.056, 1.650) 0.015	1.273 (1.014, 1.597) 0.038

OR, odds ratio.

95% CI: 95% confidence interval.

Model 1: no covariates were adjusted.

Model 2: age, gender, and race were adjusted.

Model 3: adjusted for age, gender, race, smoking status, physical activity, diabetes, hypertension, cardiovascular disease, BMI, Scr, and eGFR.

**Table 4:** The association between TyG index and UACR.

UACR	OR (95%CI) P value				
Continuous	Model 1	Model 2	Model 3	additionally adjusted for log (HOMA-IR)	additionally adjusted for metabolic syndrome
TyG index	48.614 (39.784, 57.443) <0.001	44.854 (35.497, 54.211) <0.001	28.455 (19.064, 37.846) <0.001	27.699 (17.352, 38.046) <0.001	30.348 (19.940, 40.755) <0.001
Categories					
Quartile I	1.00	1.00	1.00	1.00	1.00
Quartile II	9.474 (-7.944, 26.891) 0.286	2.632 (-15.018, 20.281) 0.770	-2.563 (-18.896, 13.769) 0.758	-4.470 (-20.961, 12.020) 0.595	-2.554 (-18.905, 13.796) 0.759
Quartile III	18.908 (1.513, 36.303) 0.033	9.161 (-8.852, 27.173) 0.318	3.249 (-13.707, 20.204) 0.707	-0.057 (-17.469, 17.354) 0.995	3.281 (-13.880, 20.441) 0.708
Quartile IV	73.236 (55.831, 90.642) <0.001	61.826 (43.435, 80.217) <0.001	28.239 (10.111, 46.367) 0.002	22.075 (2.499, 41.651) 0.027	28.346 (8.178, 48.514) 0.006

OR, odds ratio.

95% CI: 95% confidence interval.

Model 1: no covariates were adjusted.

Model 2: age, gender, and race were adjusted.

Model 3: adjusted for age, gender, race, smoking status, physical activity, diabetes, hypertension, cardiovascular disease, BMI, Scr, and eGFR.

### 3.4 Correlation of TyG index with clinical parameters

Weighted Pearson correlation analysis suggested that the TyG index was consistently positively correlated with age, gender, race, smoking status, hypertension, diabetes, cardiovascular disease, WC, FPG, TG, TC, LDL-c, Scr, urinary albumin, UACR, albuminuria, and negatively correlated with HDL-c, urinary creatinine, and eGFR, whether unadjusted, or adjusted for log (HOMA-IR), metabolic syndrome (***P*** <0.001) (**Table 5**).

**Table 5:** Correlation of TyG index with other parameters in the whole study population.

	Non-adjusted	adjusted for log (HOMA-IR)	adjusted for metabolic syndrome
log (HOMA-IR)	0.523**	–	0.349**
Metabolic syndrome	0.552**	0.399**	–
Age	0.240**	0.217**	0.118**
Gender	0.138**	0.150**	0.188**
Race	0.025*	0.083**	0.071**
Smoking status	0.110**	0.140**	0.092**
Physical activity	0.032**	0.017	0.023*
Hypertension	0.205**	0.114**	0.027**
Diabetes	0.329**	0.203**	0.177**
Cardiovascular disease	0.121**	0.073**	0.047**
BMI (kg/m^2^)	0.264**	-0.028**	0.042**
WC (cm^2^)	0.353**	0.079**	0.115**
FPG (mg/dL)	0.547**	0.393**	0.451**
FIns (µIU/ml)	0.266**	-0.138**	0.147**
TG (mg/dL)	0.750**	0.737**	0.701**
TC (mg/dL)	0.324**	0.374**	0.339**
LDL-c (mg/dL)	0.196**	0.211**	0.199**
HDL-c (mg/dL)	-0.464**	-0.335**	-0.331**
Scr (mg/dl)	0.066**	0.068**	0.044**
eGFR (ml/min/1.73 m2)	-0.130**	-0.136**	-0.063**
Urinary albumin (mg/L)	0.097**	0.064**	0.064**
Urinary creatinine (mg/dl)	-0.033**	-0.087**	-0.036**
UACR (mg/g)	0.108**	0.082**	0.073**
Albuminuria	0.155**	0.105**	0.074**

*P < 0.05; **P < 0.01.

### 3.5 Multivariate logistic regression models of albuminuria

**​**After removing covariates with VIF greater than 5, we performed a multivariate logistic regression analysis (**Table 6**). The independent variables included the TyG index, log (HOMA-IR), metabolic syndrome, age, gender, race, smoking status, physical activity, hypertension, diabetes, cardiovascular disease, BMI, Scr, and eGFR. The dependent variable was albuminuria. The results showed that TyG index [OR (95%CI): 1.283 (1.138-1.446), ***P*** <0.001] was more strongly associated with albuminuria than log (HOMA-IR) [OR (95%CI): 1.138 (0.908-1.426), ***P*** =0.263] and metabolic syndrome [OR (95%CI): 1.213 (1.024-1.438), ***P*** <0.05].

**Table 6:** Multivariate logistic regression models of albuminuria.

	OR	95%CI lower	95%CI upper	*P* value
TyG index	1.283	1.138	1.446	<0.001
Log (HOMA-IR)	1.138	0.908	1.426	0.263
Metabolic syndrome (vs. no)	1.213	1.024	1.438	0.026
Age	1.028	1.023	1.034	<0.001
Gender (vs. male)	0.474	0.400	0.562	<0.001
Race (vs. Mexican American)				
Other Hispanic	0.658	0.524	0.826	0.000
Non-Hispanic White	0.728	0.593	0.895	0.003
Non-Hispanic Black	0.721	0.554	0.939	0.015
Other Races	0.904	0.712	1.147	0.407
Smoking status (vs. never)				
Former	1.072	0.913	1.257	0.396
Current	1.307	1.097	1.558	0.003
Physical activity (vs. vigorous)				
Moderate	1.061	0.857	1.314	0.587
Less than moderate	1.056	0.880	1.268	0.557
Hypertension (vs. no)	1.673	1.441	1.942	<0.001
Diabetes (vs. no)	1.954	1.656	2.305	<0.001
Cardiovascular disease (vs. no)	1.607	1.350	1.912	<0.001
BMI (kg/m^2^)	0.998	0.987	1.009	0.702
Scr (mg/dl)	2.184	1.699	2.806	<0.001
eGFR (ml/min/1.73 m2)	1.025	1.019	1.030	<0.001

### 3.6 Evaluation of the Impact of TyG index on albuminuria

**Figure 1:**
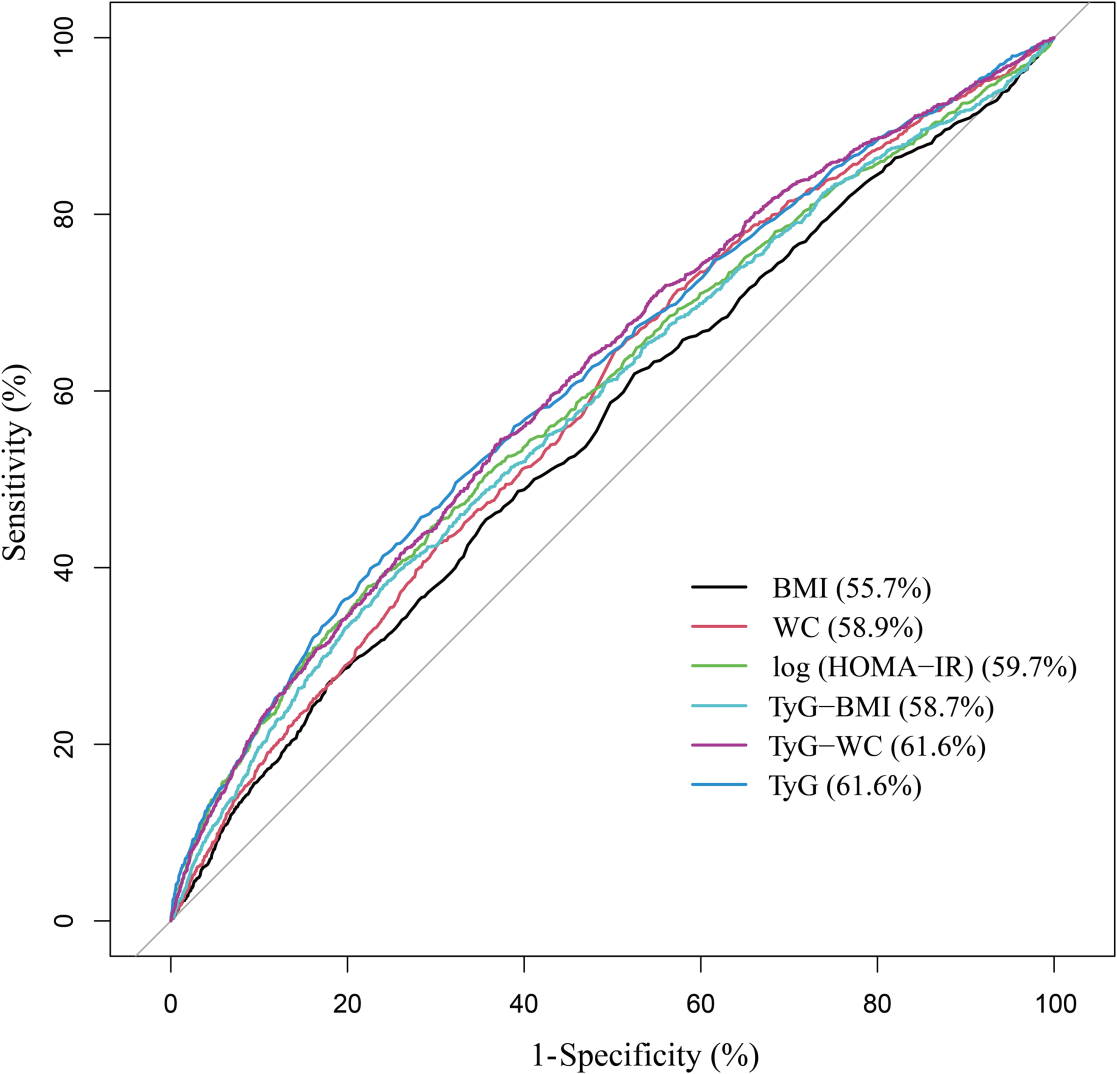
ROC analysis.

As **Figure 1** shows the performance for evaluating the endpoint among TyG index, TyG-derived indices, and log (HOMA-IR) for albuminuria risk, the AUC of the marker is as follows: BMI 0.557, WC 0.589, log (HOMA-IR) 0.597, TyG-BMI (TyG × BMI) 0.587, TyG-WC (TyG ×WC) 0.616, and TyG 0.616.

### 3.7 Subgroup analysis

**Figure 2:**
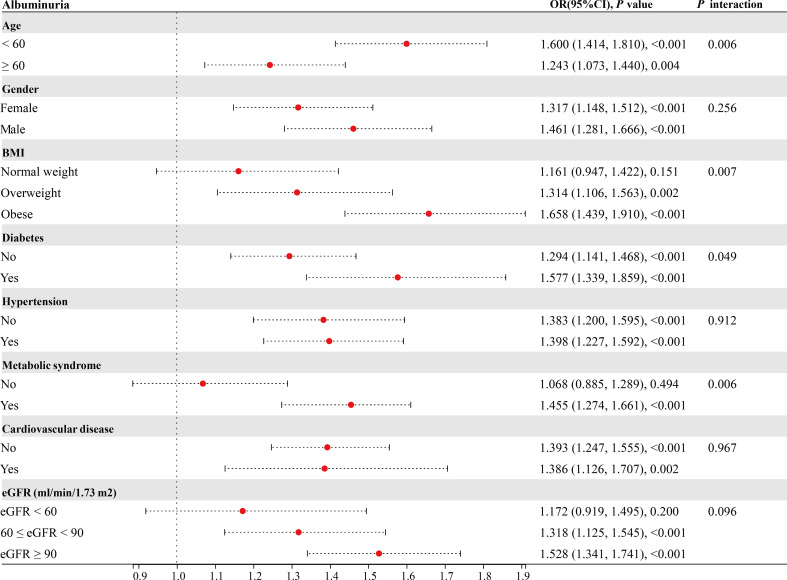
Subgroup analysis.

Our subgroup analysis showed that the degree of association between TyG index levels and albuminuria was inconsistent across populations. The interaction test suggested that the relationship between TyG index and albuminuria was influenced by age (<60/≥60), BMI (normal weight/overweight/obese), diabetes (yes/no), and metabolic syndrome (yes/no) stratification (***P*** <0.05). Age < 60, overweight/obesity, diabetes, and metabolic syndrome may be effector modifiers (**Figure 2**).

## 4 Discussion

In this cross-sectional study of 9872 participants, we found that TyG index is a valid biomarker of metabolic dysfunction with relevancy to albuminuria in the United States population. Its association with albuminuria appears to be largely independent of insulin resistance.

Insulin resistance is consistently associated with increased urinary albumin excretion (37, 38). Podocytes are thought to be the initial step in the development of albuminuria, and insulin signaling in podocytes appears to be important for maintaining the integrity of the glomerular filtration barrier (39, 40). Insulin resistance can also induce adipose tissue inflammation to activate and release proinflammatory cytokines such as IL-6 and TNF-α, which induce endothelial dysfunction, leading to albuminuria and impaired renal function (41, 42). Several previous studies have also described the association between TyG and albuminuria from this perspective (28, 30, 43). However, TyG index continued to be associated with albuminuria after mutual adjustment of HOMA-IR, suggesting that the mechanism of albuminuria induced by insulin resistance could not fully explain the effect of TyG index. This finding was also hinted at a previous longitudinal study of the Chinese population. Participants in the high TyG index, low HOMA-IR group had a higher risk of new-onset albuminuria than participants in the low TyG index, low HOMA-IR group (44). Additionally, the ROC curves showed that the evaluation value of the TyG index for albuminuria was significantly higher than log (HOMA-IR), while the TyG-derived indices did not greatly improve. The TyG index has been extensively studied and validated as a marker of insulin resistance and metabolic abnormalities, unlike other TyG-derived indices. However, the TyG-WC index may also be valuable since it considers waist circumference, an essential measure of central obesity.

Our study further discovered that TyG’s link to albuminuria persisted even after taking metabolic syndrome into account. This highlights the additive value of the TyG index in the investigation of metabolic risk factors for albuminuria. It may be more beneficial to use continuous rather than dichotomous measures of metabolic syndrome. At the same time, the results of mutual correction based on multivariate logistic regression can also be seen that the correlation between TyG index and albuminuria was stronger than that between HOMA-IR, metabolic syndrome, and albuminuria.

Further stratified subgroup analyses and interaction test suggested that the relationship between TyG index and albuminuria is of greater concern in age<60, overweight/obese, diabetic, and metabolic syndrome patients, at least in the United States population. Hypertension can worsen kidney injury and cause albuminuria, an important marker for cardiovascular events. Previous studies have also suggested a bidirectional association between hypertension and metabolic dysfunction, such as NAFLD (45). However, we did not find that hypertension or cardiovascular disease influenced the magnitude of the association between TyG index and albuminuria. This may suggest that the TyG index is more directly reflect the effects of metabolic dysfunction on albuminuria and is more valuable in people with metabolic dysfunction.

What is clear is that TyG index was associated with albuminuria, a relationship that was largely independent of insulin resistance represented by HOMA-IR. Future studies are needed to determine its performance as a predictive biomarker. In addition, special attention should be paid to differences in subgroups based on age, BMI, diabetes, and metabolic syndrome. There are some limitations in this study. First, due to the cross-sectional design of this study, a prospective study with large sample size is needed to clarify the causal relationship. Second, although we adjusted for some potential covariates, we could not completely exclude the influence of other possible confounders. Finally, the stratification of other subgroups was not considered, such as different types of diabetes, NAFLD, etc.

## 5 Conclusion

In a nationally representative study of adults aged ≥20, the TyG index was associated with albuminuria. These findings cannot be fully explained by the insulin resistance or the dichotomous definition of metabolic syndrome. This metric shows promise as an epidemiological tool for quantifying the role of metabolic dysfunction in albuminuria and possibly for predictive value.

## Data availability statement

Publicly available datasets were analyzed in this study. This data can be found here: https://www.cdc.gov/nchs/nhanes/.

## Ethics statement

The studies involving human participants were reviewed and approved by National Center for Health Statistics (NCHS) Research Ethics Review Board. The patients/participants provided their written informed consent to participate in this study.

## Author contributions

All authors listed have made a substantial, direct, and intellectual contribution to the work and approved it for publication.
